# Physician’s Leisure Library

**Published:** 1889-07

**Authors:** 


PHYSICIAN'S LEISURE LIBRARY.—We have received the following copies of this useful and instructive series of publications which have not been specially noticed heretofore; to wit:

Pulmonary tuberculosis—its etiology, symptomalology and therapeutics by Prof. Dr. H. Von Ziemessen, Director of Medical Clinic at Munich. Translated by David J. Doherty, A. M., M. D., Instructor in Chicago Policlinic.

A Treatise on Hysteria and epilepsy, with some concluding observations on epileptic insomnia by J. Leonard Corning, M. A., M. D., consultor in nervous diseases to St Francis Hospital, etc., etc.

Clinical lectures on certain diseases of the nervous system, by Prof. J. M. Charcot, Prof. of the Faculty of Medicine, Paris, etc. Translated by E. P. Hurd, M. D.

A Treatise on Hernia. The Radical cure by the use of buried antiseptic animal suture, by Henry O. Marcy, A. M., M. D., L. L. D., Surgeon to Private Hospital for Women, Cambridge, etc., etc.

These works copyrighted and published by Geo. S. Davis, Detroit, convey much useful and instructive reading to the profession in a cheap form. Price for each work in paper, 25cts., in cloth, 50cts.

